# Novel micro-reactor flow cell for investigation of model catalysts using *in situ* grazing-incidence X-ray scattering

**DOI:** 10.1107/S1600577516001387

**Published:** 2016-02-18

**Authors:** Jan Kehres, Thomas Pedersen, Federico Masini, Jens Wenzel Andreasen, Martin Meedom Nielsen, Ana Diaz, Jane Hvolbæk Nielsen, Ole Hansen, Ib Chorkendorff

**Affiliations:** a Center for Individual Nanoparticle Functionality, Department of Physics, Technical University of Denmark (DTU), Fysikvej, 2800 Kgs Lyngby, Denmark; b Department of Micro and Nanotechnology, Technical University of Denmark (DTU), Ørsteds Plads, 2800 Kgs Lyngby, Denmark; c Department of Energy Conversion and Storage, Technical University of Denmark (DTU), Frederiksborgvej 399, PO Box 49, 4000 Roskilde, Denmark; d Department of Physics, Technical University of Denmark (DTU), Fysikvej, 2800 Kgs Lyngby, Denmark; e Paul Scherrer Institute, 5232 Villingen PSI, Switzerland

**Keywords:** *in situ*, GISAXS, GIWAXS, micro-reactor

## Abstract

The design, fabrication and performance of a novel and highly sensitive micro-reactor device for performing *in situ* grazing-incidence X-ray scattering experiments of model catalyst systems is presented. The design of the reaction chamber, etched in silicon on insulator (SIO), permits grazing-incidence small-angle X-ray scattering (GISAXS) in transmission through 10 µm-thick entrance and exit windows by using micro-focused beams. An additional thinning of the Pyrex glass reactor lid allows simultaneous acquisition of the grazing-incidence wide-angle X-ray scattering (GIWAXS). *In situ* experiments at synchrotron facilities are performed utilizing the micro-reactor and a designed transportable gas feed and analysis system. The feasibility of simultaneous *in situ* GISAXS/GIWAXS experiments in the novel micro-reactor flow cell was confirmed with CO oxidation over mass-selected Ru nanoparticles.

## Abstract 2

The design and performance of a novel micro-reactor *in situ* flow cell permitting investigation of model catalysts with grazing-incidence small- and wide-angle X-ray scattering is presented.

## 1. Introduction   

Clausen *et al.* made a breakthrough approximately 25 years ago with the development of a flow cell for investigation of heterogeneous catalysts under conditions comparable with large-scale industrial processes using powder diffraction at synchrotron facilities (Clausen *et al.*, 1991 ▸). Since then, a multitude of different flow cells for *in situ* investigation of catalyst materials with single techniques such as X-ray absorption spectroscopy (XAS) (Girardon *et al.*, 2005 ▸), small-angle X-ray scattering (SAXS) (Andreasen *et al.*, 2003 ▸; Høydalsvik *et al.*, 2014 ▸), wide-angle X-ray scattering (WAXS) (Andrieux *et al.*, 2014 ▸) and combinations of these techniques such as SAXS/WAXS (Kehres *et al.*, 2010 ▸, 2012 ▸) or XAS/WAXS (Hannemann *et al.*, 2007 ▸) have been developed.

*In situ* investigations of heterogeneous catalysts applying these techniques are often performed utilizing powdered samples to investigate the fundamental relations between activity, particle size and size distribution, morphology, crystallinity and oxidation state. The heterogeneous catalyst samples for the *in situ* investigations are usually synthesized by conventional and also industrial applied routes, such as incipient wetness impregnation of the substrate or co-precipitation, yielding random orientation and broad particle size distributions. The complexity of such catalyst samples limits the extractable amount of information.

The amount of information that can be gathered about catalyst particles during *in situ* experiments can be substantially increased when applying SAXS and WAXS in grazing-incidence geometry (GISAXS and GIWAXS). Because of the low penetration depth of the X-rays in the bulk material at incidence angles close to the critical angle of the substrate, the patterns display nearly solely scattering from the catalyst nanoparticles. Since the particles are deposited on flat surfaces, *in situ* investigation of their shape is facilitated, ranging from determination of aspect ratios for randomly oriented particles on amorphous substrates (Winans *et al.*, 2006 ▸; Wyrzgol *et al.*, 2010 ▸) to changes of the faceting (Nolte *et al.*, 2008 ▸; Molina *et al.*, 2011 ▸) and resolving the average morphology for particles having epitaxial orientation on the support (Renaud *et al.*, 2003 ▸). *In situ* investigations of idealized systems are indispensable to gain fundamental understanding of catalytic reactions, *i.e.* to resolve relations between chemical reactivity and particle morphology.

Such idealized systems can be produced by deposition of gas-aggregated and mass-selected metal nanoparticles (Issendorff & Palmer, 1999 ▸; Molina *et al.*, 2011 ▸; Nielsen *et al.*, 2010 ▸; Vajda *et al.*, 2006 ▸; Winans *et al.*, 2006 ▸) on a flat substrate. The model catalysts, prepared by this method, can be investigated in elaborated *in situ* hotplate setups with grazing-incidence X-ray techniques. Simultaneously monitoring the gas atmosphere can be performed to follow the conversion. Huge advances in the development of a suitable *in situ* hotplate flow cell for studying model catalysts at ambient pressure, and thereby under conditions closer to large-scale industrial processes than the usual performed experiments in ultrahigh vacuum (UHV), with GISAXS/GIXAS were made by Lee *et al.* (2011 ▸). The optimized design of this *in situ* cell results in a small volume and hence in a high sensitivity for analysis of the catalytic conversion, water cooling and surface coating of all metal parts suppressing possible side reactions. Pioneering work in developing an *in situ* hotplate flow cell permitting grazing-incidence X-ray experiments in a pressure range between 100 and 1200 mbar was performed and reported by Van Rijn *et al.* (2010 ▸). Besides its very small volume, the cell presented by Van Rijn *et al.* (2010 ▸) has a complete UHV sample preparation chamber, including mass spectrometer, sample cleaning equipment and a metal evaporator for the model catalyst preparation. Contamination of the prepared samples due to the sample transfer in air can therefore be avoided.

A higher sensitivity and faster equilibrium of the gas phase after changing the test conditions can be reached by decreasing the flow cell volume further. Elaborate micro-structured reaction chambers with very small volumes can be produced by photolithography and etching of silicon wafers and the chambers can be closed tight by anodic bonding of glass. A silicon/glass micro-reactor device, allowing the complete exchange of the reaction volume in a time on the order of seconds, was developed at DTU (Henriksen *et al.*, 2009 ▸). This micro-reactor platform is frequently used in the Department of Physics at DTU to investigate chemical reactions over model (Andersen *et al.*, 2012 ▸; Henriksen *et al.*, 2009 ▸; Jensen *et al.*, 2013 ▸) and photo-catalysts (Dionigi *et al.*, 2012 ▸; Nielsen *et al.*, 2012 ▸; In *et al.*, 2011 ▸; Vesborg *et al.*, 2010*a* ▸).

**Figure 1:**
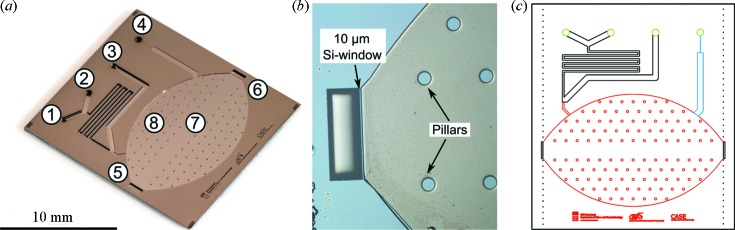
(*a*) Etched structure of an *in situ* grazing-incidence X-ray scattering micro-reactor flow cell before anodic bonding of the Pyrex lid and dicing to reveal the silicon windows. The gas inlets (1) and (2), the bypass (3), the capillary to the mass spectrometer (4), 10 µm-thick entrance and exit windows (5) and (6), beam path without supporting pillars (7) and supporting pillars to avoid reactor chamber collapse when working at lower than ambient pressure (8) are labelled on the image. (*b*) Close-up of the 10 µm-thick Si entrance window and pillar structure of an anodic-bonded closed reactor template before etching and dicing. (*c*) Schematic representation of the micro-reactor device.

We recently developed the micro-reactor further to permit the opportunity of performing *in situ* GISAXS/GIWAXS experiments at micro-focus beamlines (see Fig. 1*a* ▸) in addition to continuous monitoring of the gas phase by mass spectroscopy. Our *in situ* micro-reactor flow cell, in connection with the aforementioned methods of analysis, provides an ideal framework for determining the physical state of the nanoparticles, *i.e.* morphology, phase composition and crystallite size, and establishing correlations between the nanoparticle state and catalytic activity. A silicon/glass micro-reactor flow cell for mixing liquids in a T-junction, manufactured by photolithography and etching, for *in situ* SAXS/WAXS was very recently developed and its application was demonstrated by following a crystallization process by Beuvier *et al.* (2015 ▸).

In the work presented here we demonstrate the application of the *in situ* micro-reactor flow cell for the investigation of dynamical changes of mass-selected Ru nanoparticles during CO oxidation under net oxidizing conditions using a combination of GISAXS and GIWAXS and simultaneous analysis of the product gas by mass spectroscopy.

## 2. Description of the system   

### 2.1. The micro-reactor   

The general design of the micro-reactor flow cell was reported by Henriksen *et al.* (2009 ▸). Two reactant gases, or reactant gas mixtures, can be applied simultaneously and mixed in the micro-reactor chip. The complete product gas leaves the micro-reactor through a flow-limiting capillary, which is etched in the chip, into a quadrupole mass spectrometer (QMS). Excess gas from the mixing channels, not entering the reaction chamber, leaves the micro-reactor device through a bypass. To permit the possibility of performing *in situ* GISAXS the micro-reactor cell was re-designed. The reaction chamber has the shape of an ellipse truncated on the major axis with a 10 µm-thick and 1500 µm-wide entrance window for the incident beam and an exit window of the same dimensions for the GISAXS. The chamber height of the *in situ* micro-reactor was increased to 50 µm to permit incidence angles α_i_ up to 0.25° considering experiments with a micro-focused beam with a beam height of maximum about 10 µm. The supporting pillar structure, that prevents collapse of the micro-reactor chamber during pumping, was removed to a 1500 µm-wide region along the major axis of the cell (see Fig. 1*b*) ▸.

The capillary connecting the reaction chamber and the mass spectrometer was dimensioned by taking the limiting pumping capacity of the vacuum system of the mass spectrometer into account and retaining a pressure of about 10^−7^ mbar around the QMS. Flows of about 6 × 10^15^ molecules s^−1^ through the reaction chamber are obtained with capillary dimensions of 1500 µm × 5.5 µm × 10.0 µm (length × width × depth). With a reaction chamber volume of 5.8 × 10^−3^ cm^3^, this corresponds to a residence time of only about 23 s for the reactant gas. In principle the micro-reactor chip can be operated in a pressure range from UHV to about 1.8 bar, and a delamination of the 20 µm-thick reactor lid was observed at pressures of ∼2 bar; however, experiments in constant gas flow are intended to be performed at pressures between 0.1 and 1.2 bar.

The X-ray transparent micro-reactors were fabricated on 4"-diameter silicon on insulator (SOI) wafers with a 50 µm Si device layer, a 1 µm buried oxide layer and a 500 µm Si handle wafer. The reactor was created by a series of UV photolithograpy, reactive ion etching (RIE) and deep reactive ion etching (DRIE) processes. For all photolithography processes an AZ5214E photoresist with a thickness of 1.5 µm was used. The RIE/DRIE processes were all performed using an SPTS Pegasus tool. A top-down view of the reactor chip is given in Fig. 1(*c*) ▸.

The first step is to define and etch the flow limiting capillary, shown in blue in Fig. 1(*c*) ▸. The pattern is defined by photolithography and etched to a depth of 10 µm by RIE after which the photoresist is removed with acetone. Hereby the 10 µm-deep, 5.5 µm-wide and 1500 µm-long flow limiting capillary is formed.

The reactor chamber itself was 50 µm deep, and the main gas channels were 250 µm deep. Creating such deep structures in two separate photolithography- and etch-processes is difficult so we used a technique involving a latent oxide mask described in the following. First, a 300 nm thermal oxide is grown on the substrate. Then the reactor pattern, shown in red in Fig. 1(*c*) ▸, is etched through the 300 nm oxide layer using a photolithography and buffered hydrofluoric acid (bHF) etch process after which the photoresist is removed with acetone. Then a new photolithography process is carried out to define the gas channel pattern, shown in black in Fig. 1(*c*) ▸. The pattern is first etched through the 300 nm oxide with bHF and then etched (using DRIE) through the 50 µm silicon device layer until the buried oxide is reached. At this point it is necessary to etch through the 1 µm buried oxide with bHF before proceeding with the DRIE process, etching another 150 µm to a total etched depth of 200 µm. After this, the photoresist layer is removed with acetone, and another 50 µm silicon is etched using the DRIE process. This etch uses the 300 nm oxide as an etch mask and the reactor area, shown in red in Fig. 1(*c*) ▸, is also exposed and etched out until the buried oxide is reached. In this way the reactor area is etched to a depth of 50 µm and the gas channels are etched to a final depth of 250 µm. The remaining part of the 300 nm oxide layer and the buried oxide exposed in the bottom of the reactor is etched away with bHF. The final process is to etch out the inlet/outlet gas holes through the wafer, shown in green in Fig. 1(*c*) ▸. This is done by performing a photolithography and DRIE process on the back of the wafer. Finally, a 50 nm-thick thermal oxide is grown; this serves mainly as an inert support layer onto which the catalyst particles can be deposited.

**Figure 2:**
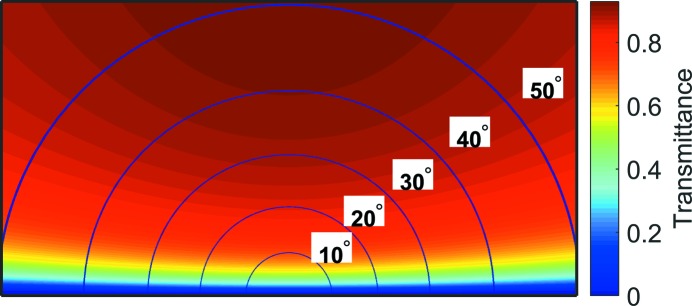
Angle-dependent transmission through a 20 µm reactor lid calculated for an X-ray energy of 11.2 keV.

After deposition of the catalyst nanoparticles, the reactor is sealed off by anodic bonding of a 200 µm-thick Pyrex glass using locally cooled anodic bonding (Vesborg *et al.*, 2010*b* ▸). This glass is then thinned down to 20 µm in a timed HF etch to allow higher transmission during GIWAXS measurements. After the thinning of the Pyrex lid, the sides of the reactor chip are diced off with a diamond blade, along the dotted lines shown in Fig. 1(*c*) ▸, to reveal the 10 µm-thick silicon windows. The calculated transmission through the reactor lid for at a photon energy of 11.2 keV is illustrated in Fig. 2 ▸ as a function of the scattering angle. The reaction chamber of the *in situ* micro-reactor cell covers an angular range of 2θ = 0–5° in the substrate plane and about α_f_ = 0–0.30° for a 50 µm-high reaction chamber for out-of-plane GISAXS, considering an incident beam with a height of 10 µm. An X-ray transmission of more than 0.5 for the GIWAXS is obtained at an out-of-plane angle of α_f_ > 5°. Analysis of the out-of-plane GISAXS of catalyst nanoparticles in the micro-reactors flow cell is not feasible because of the limited resolvable scattering vector perpendicular to the substrate (*q*_*z*_). The extractable information is therefore restricted to the characteristic lengths of the lateral correlations, *e.g.* lateral particle diameter and inter-particle distance. For a complete understanding of the structural changes of the nanoparticles occurring during catalytic reactions resolving the particle aspect ratio *H*/*R* (height/radius) to follow the evolution of the particle height *H*, a larger accessible exit angle will be required. We are currently investigating options to redesign the micro-reactor to permit a higher exit angle by increasing the chamber height or/and decreasing the chamber width.

### 2.2. Gas system   

**Figure 3:**
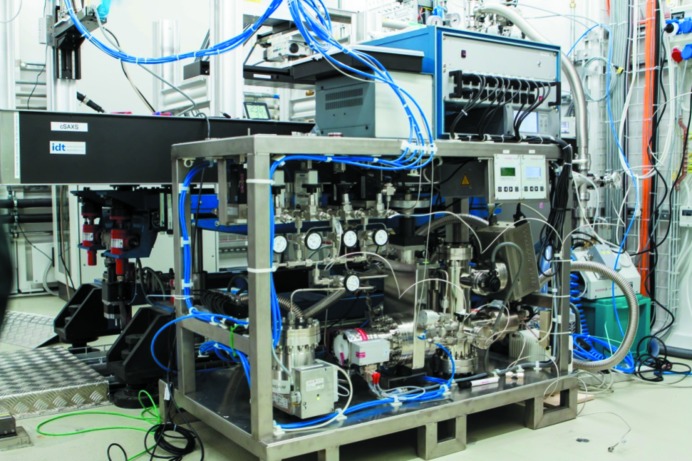
Photograph of the transportable gas system for use at synchrotron facilities during an *in situ* CO oxidation experiment at the cSAXS beamline at the Swiss Light Source at the Paul Scherrer Institute in Switzerland.

The transportable gas system, which was recently developed for use with different flow cells at synchrotron radiation facilities, was designed with a base similar to a euro-pallet and can be moved with any pallet or fork lift truck (see Fig. 3 ▸).

**Figure 4:**
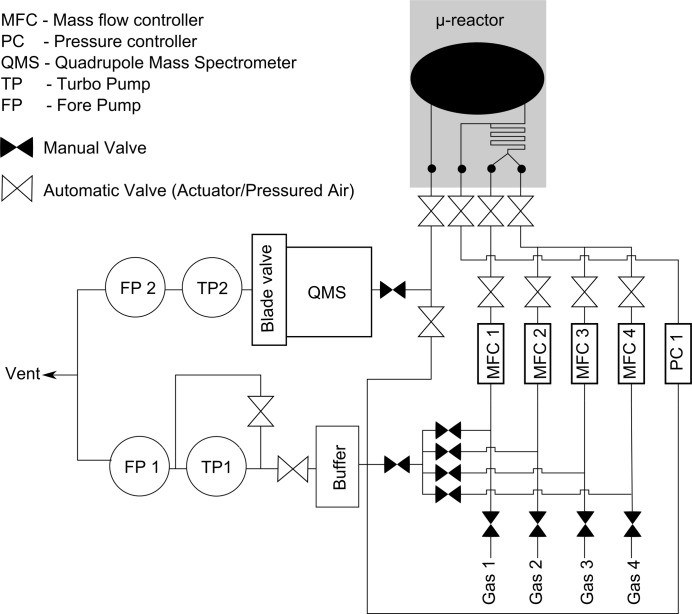
Flow scheme of the transportable gas system, connected to the *in situ* X-ray scattering micro-reactor cell.

All lines of the gas handling system are manufactured from 1/4" welded tubes with VCR^®^ (Swagelok) connections. The buffer volume and the analysis chamber are made from stainless steel tubes with CF flanges sealed with copper gaskets. Pneumatic valves with a solenoid manifold are installed for all gas connections that are frequently switched to allow remote control without interrupting the data acquisition during an experiment by entering the hutch. The *in situ* micro-reactor flow cell is connected to the transportable gas system with a slightly modified manifold of the same type as described by Henriksen *et al.* (2009 ▸); welded 1/8" tubes with VCR^®^ tubes are used for the connection to obtain flexibility. A flow scheme of the gas system is shown in Fig. 4 ▸.

## 3. Experimental   

**Figure 5:**
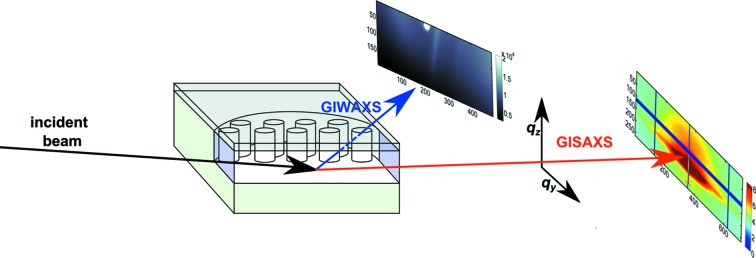
Schematic representation of the geometry of the *in situ* GISAXS/GIWAXS experiment with the incident beam entering the reactor chamber under an incident angle of α_i_ = 0.2° through the 10 µm-thick Si entrance window in the middle of the reaction chamber where no support pillars are blocking the incident or scattered beam. The in-plane GISAXS and the out-of-plane GISAXS up to an angle of α_f_ = 0.30° are transmitted through the 10 µm-thick Si exit window. The GIWAXS is transmitted through the lid which has been thinned to approximately 20 µm.

The *in situ* GISAXS/GIWAXS experiments were performed at the cSAXS beamline at the Swiss Light Source (SLS) at the Paul Scherrer Institute in Villigen, Switzerland. GISAXS data were acquired with a Pilatus 2M detector (Henrich *et al.*, 2009 ▸) with a sample-to-detector distance of 2120 mm. The GIWAXS data were captured with a Pilatus 100K module, installed 18° off-axis with respect to the direct beam, with a sample-to-detector distance of about 242 mm. The geometry of the *in situ* GISAXS/GIWAXS experiment in the micro-reactor is illustrated in Fig. 5 ▸.

The experiments were performed with a beam size of about 10 µm × 100 µm (height × width) and an X-ray energy of 11.2 keV (λ = 1.11 Å). A laser-polished yttrium-stabilized zirconia (YSZ) pill was measured at the sample position. The YSZ (111)-, (200)- and (220)-reflections were utilized to perform an angular calibration to relate each detector pixel to a certain scattering angle.

The mass-selected Ru nanoparticles were prepared using a magnetron sputter gas aggregation source (Birmingham Instruments Inc.), combined with von Issendorff time-of-flight mass filtering (Issendorff & Palmer, 1999 ▸), and deposited onto a micro-reactor mounted in a multichamber UHV system (Omicron, Multiscan Lab) with a base pressure in the low 10^−10^ mbar region. Ru nanoparticles, with a mean diameter of 10 nm, were deposited through a spherical aperture directly onto the bottom of the chamber of the micro-reactor flow cell. With this method, particle deposits corresponding to an average geometrical coverage of 10% of a layer and spot sizes of about 64 mm^2^ were prepared.

Sample heating was performed with a cylindrical resistance heater with a maximum power of 120 W attached to the Si-wafer of the *in situ* micro-reactor and a variable DC power supply. The sample temperature was determined with a type-K thermocouple attached to the Si wafer beneath the reactor and by a pyrometer (Impac, Infratherm IGA 15+) mounted on a tripod and adjusted to the sample surface, assuming an emissivity of 0.95 for the Pyrex glass lid. The pyrometer temperature was read out through a camera installed in the hutch at the cSAXS beamline. The combination of both techniques for the temperature determination was selected for the following reason: in the presented experiment a simple isolation of the micro-reactor flow cell was not possible. The determination of the specimen temperature utilizing the thermocouple was therefore observed to be influenced by disturbances from the constant air flow of the air-condition system. The measured voltage and the respectively determined temperature were observed to be extremely sensitive to how the thermocouple was in contacted with the surface. A linear response of both drawn power by the heater and measured voltage by the thermocouple with the temperature determined by the pyrometer on the surface of the reactor lid was observed and we therefore consider the measured temperature with the pyrometer as the best possible approximation of the temperature inside the reaction chamber. However, measurements of temperatures below 523 K are not possible with the type of pyrometer used for the experiments. The temperature inside the reaction chamber for *T* < 523 K was therefore estimated from the parameters obtained from linear regression analysis of the apparent temperature measured with the thermocouple and the pyrometer. A more elaborate resistive sample heating and temperature determination from Pt strips evaporated on the Si side of the micro-reactor (Andersen *et al.*, 2012 ▸) will be implemented in the near future. With the Pt strips we will achieve more precise temperature measurements and a better heat transfer, because of the direct contact with the Si part of the micro-reactor device. Isolation of the Si part of the reaction chamber will be facilitated which will result in more homogeneous heating and reduced heat dissipation.

**Figure 6:**
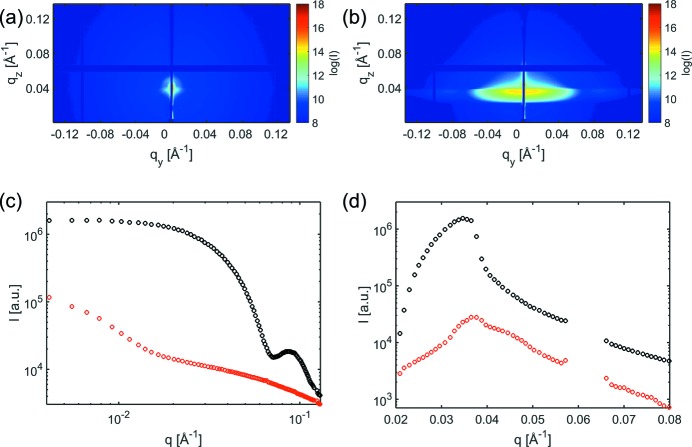
Two-dimensional GISAXS measurement of an empty reactor (*a*) and with ruthenium (Ru) nanoparticles of diameter 10 nm (*b*). Cuts along the horizontal direction at *q*_*z*_ = 0.036 Å^−1^ (*c*) and vertical direction at *q*_*y*_ = 0.01 Å^−1^ (*d*) for the empty reactor (red) and with Ru particles (black).

The two-dimensional GISAXS measurements for an empty micro-reactor and a reactor with 10% of a layer of Ru particles of diameter *d* = 10 nm acquired with an incident angle of the direct beam of α_i_ = 0.2° are shown in Figs. 6(*a*) and 6(*b*) ▸, respectively; Figs. 6(*c*) and 6(*d*) ▸ show in-plane and out-of-plane line-cuts, respectively. It can be seen that the GISAXS is dominated by scattering of the Ru particles with comparatively low contributions of scattering from the rough surface structures originating from the etching process during the manufacturing of the reactor templates.

## 4. Application: CO oxidation over Ru nanoparticles   

**Figure 7:**
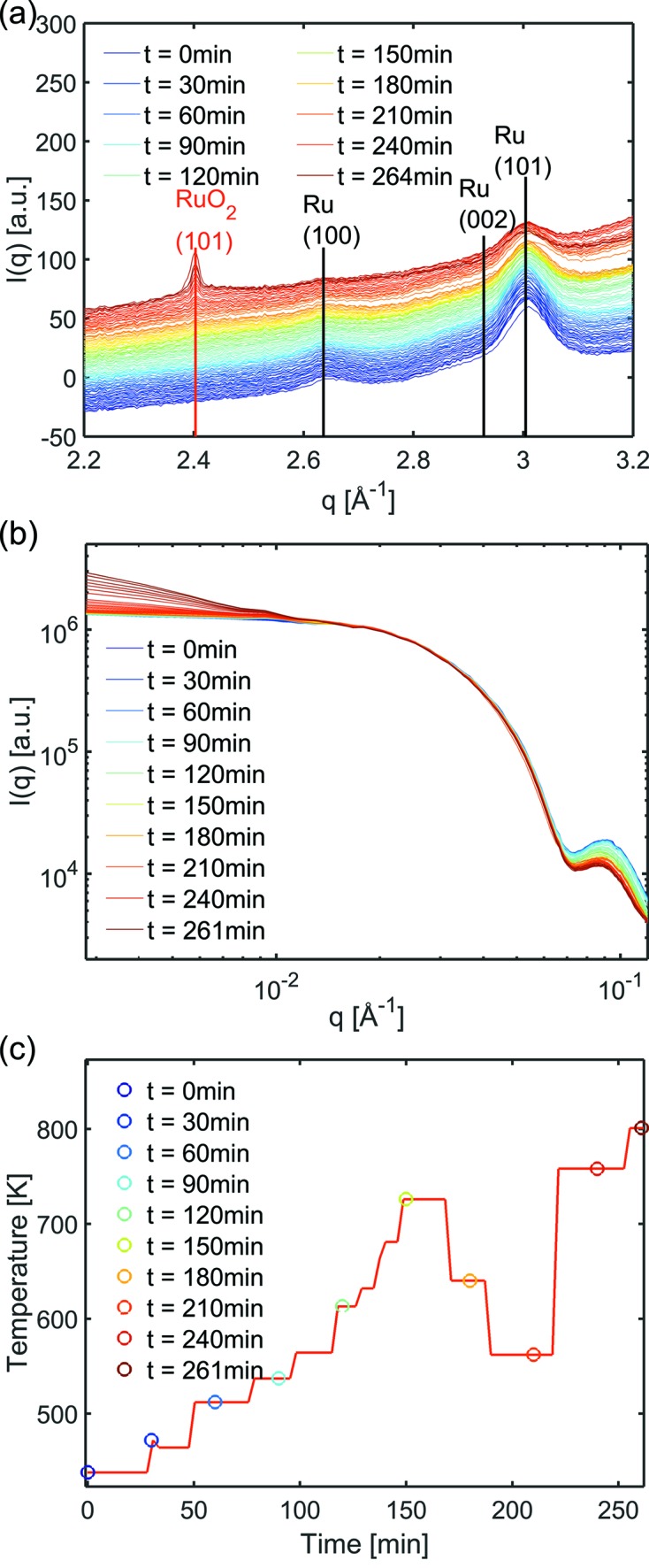
Evolution of averaged GIWAXS (*a*) and the in-plane GISAXS (*b*) at *q*_*z*_ = 0.036 Å^−1^ during the CO oxidation with time and temperature profile of the experiment (*c*). The GIWAXS data are plotted with an offset for better visibility.

To demonstrate the applicability of the *in situ* micro-reactor flow cell during reaction, CO oxidation over Ru nanoparticles was performed,
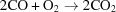
For removal of adsorbed molecules on the sample surface due to the sample handling in air and for reduction of the native RuO_2_ layer, the reactor was heated to 433 K while applying a flow of 3 ml min^−1^ H_2_ in He with *p*(H_2_) = 50 mbar at a total pressure of *p*_abs_ = 800 mbar and the sample was kept at 433 K for 15 min. For the CO oxidation a reactant gas mixture with *p*(O_2_) = 40 mbar and *p*(CO) = 20 mbar in He with a flow of 5 ml min^−1^ was applied to the sample at *p*_abs_ = 800 mbar. The evolution of the GISAXS and GIWAXS as a function of time during the first heating cycle of the CO oxidation can be observed in Fig. 7 ▸, where both the radially integrated GIWAXS (Fig. 7*a* ▸) and the in-plane GISAXS (Fig. 7*b* ▸), obtained from line-cuts *q*_*z*_ = 0.036 Å^−1^, are shown. Fig. 7(*c*) ▸ shows the applied sample temperature as a function of time.

Qualitatively, at higher temperatures and longer time exposed to the reactant gas, a decrease of the Ru(100), (002) and (101) reflections and an increase of the RuO_2_(101) reflection is observed. The in-plane GISAXS shows no visible shift of the knee-like Guinier regime (Guinier & Fournet, 1955 ▸) towards lower wavenumber *q*, indicating that the lateral dimension of the Ru particles remains nearly constant. At highest sample temperatures an increase of the scattered intensity at lowest *q* is observed indicating the formation of larger clusters by particle sintering.

**Figure 8:**
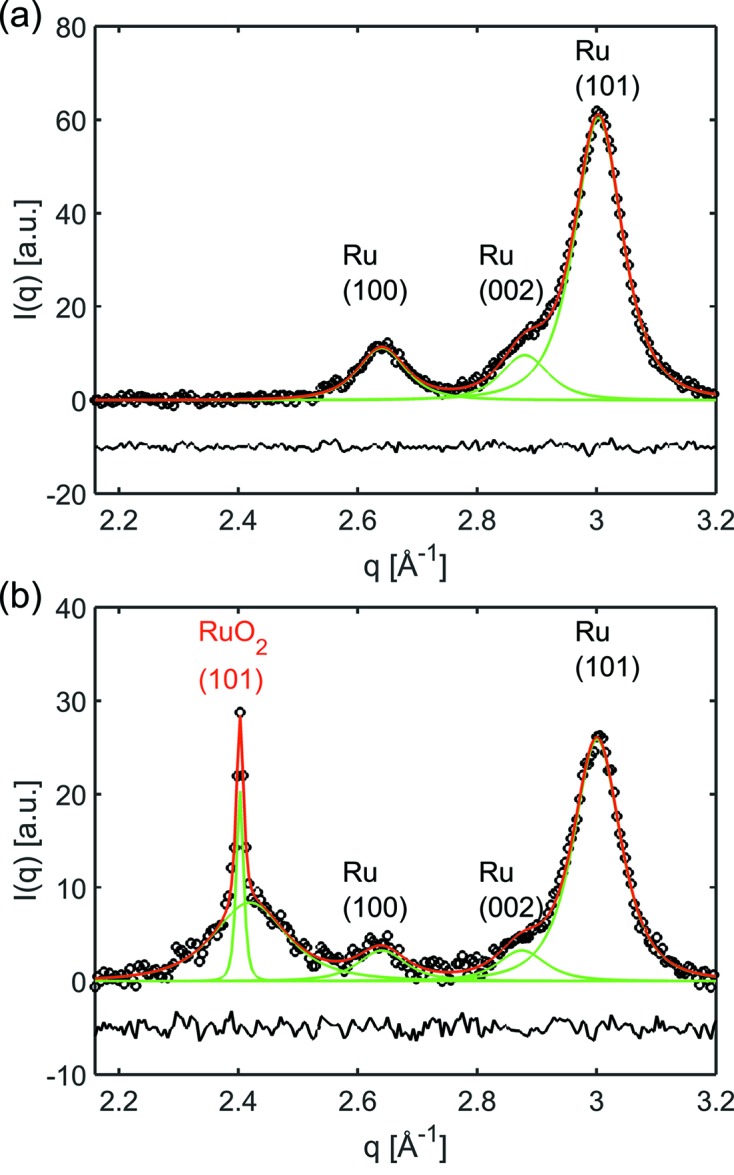
Illustration of the fit of the background-subtracted GIWAXS data of the reduced catalyst before introducing reactant gas into the micro-reactor with three Pearson VII peak profiles for the Ru(100), (002) and (101) reflections (*a*) and after the first heating cycle in reactant gas (*b*), with two additional peak profiles for the RuO_2_(101) reflection arising from small and large crystallites. The background was approximated by a sixth-order polynomial and has been subtracted in the plots.

The averaged GIWAXS data were analysed by fitting three Pearson VII peak profile functions for the Ru(100), (002) and (101) reflections. The contribution of the emerging RuO_2_ phase was taken into account by the fit of two additional Pearson VII peak profiles for the RuO_2_(101) reflection to describe the scattering of small and large RuO_2_ crystallites. The background was fitted with a sixth-order polynomial. To limit the number of free parameters, the full width at half-maximum (FWHM) of the three Ru reflections was restrained to be equal; while this is not exactly true it is a good approximation for the limited *q*-range of 0.7 Å^−1^ in which the fit was performed. The fit of the GIWAXS peaks before introducing reactant gas into the micro-reactor device and the fit after the first heating cycle in reactant gas of 1CO:2O_2_ are shown in Figs. 8(*a*) and 8(*b*) ▸, respectively. The crystallite of the fresh Ru catalyst particles before reaction was refined with *d* = 10 nm, determined from the FWHM of the Ru(101) peak using the Scherrer equation with a pre-factor for spherical crystallite shape. The width of the reflection was corrected for instrumental broadening, estimated from the experimental geometry.

**Figure 9:**
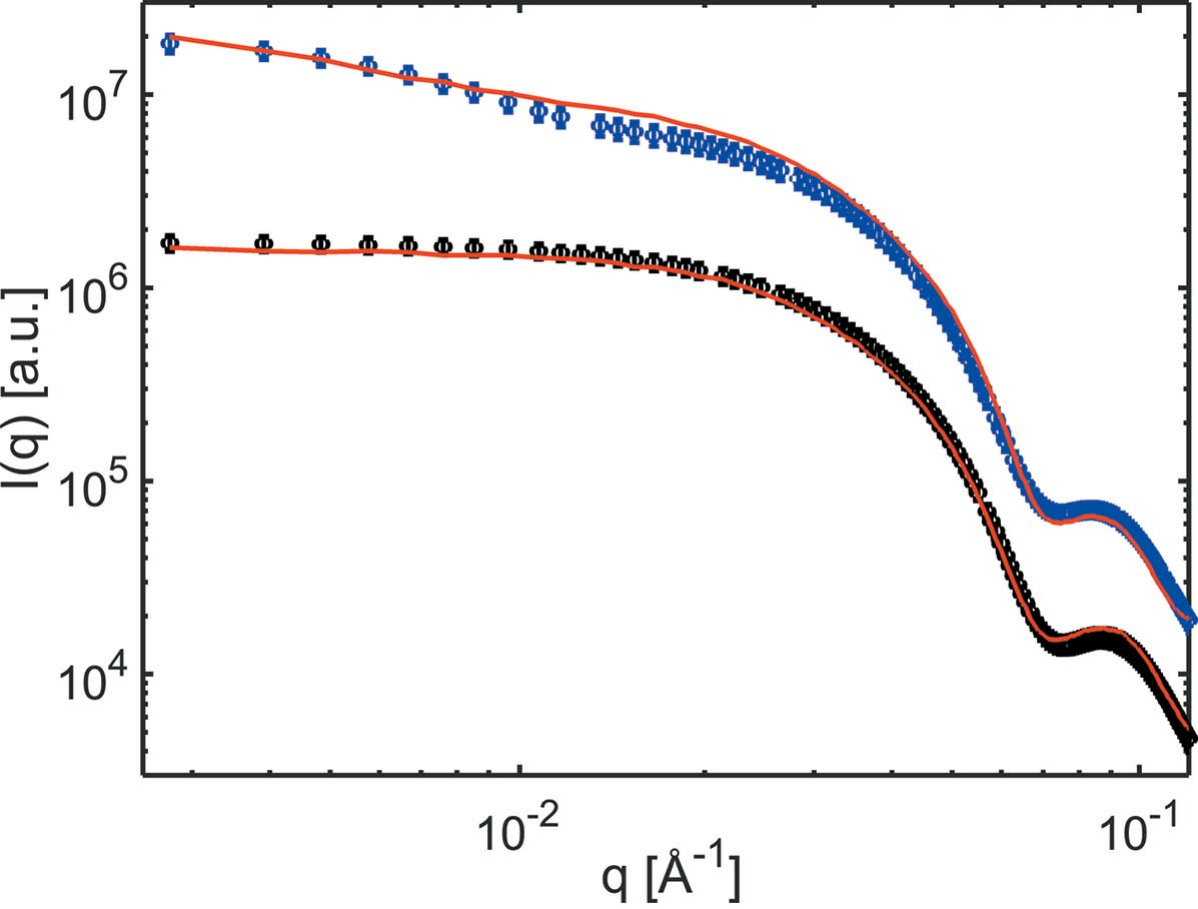
Horizontal one-dimensional GISAXS profiles (circles) and fit with a form factor for on-the-base truncated sphere (red line) prior to introducing reactant gas and heating (black circles) and after the first heating cycle to a temperature of 758 K (blue circles). The data are plotted with an offset for better visibility.

The in-plane line-cuts of the GISAXS patterns were analysed using the *IsGISAXS* software (Lazzari, 2002 ▸) in the framework of the distorted-wave Born approximation (DWBA). The average particle shape was approximated with a form factor of a sphere truncated on the base with an aspect ratio *H*/*R* (height/radius) of 1.8, as observed in previous experiments from analysis of Ru particles deposited with the same cluster source directly onto SiO_2_/Si wafers. Resolving *H*/*R* of the Ru particles directly from the GISAXS of the micro-reactor in the current design is not feasible due to the limited maximum exit angle of about 0.3°. For the analysis a Gaussian particle size distribution was assumed. For the fresh Ru nanoparticles we obtained a lateral mean particle diameter of *D* = 11.5 nm and a standard deviation of σ_D_/*D* = 0.16 corresponding to a standard deviation in particle size of ±1.9 nm. In-plane line-cuts of the GISAXS together with the fits are shown in Fig. 9 ▸. The particle size determined from analysis of the GISAXS data agrees well with the crystallite size obtained from size-broadening analysis from the GIWAXS, suggesting the Ru particles to be single crystalline.

**Figure 10:**
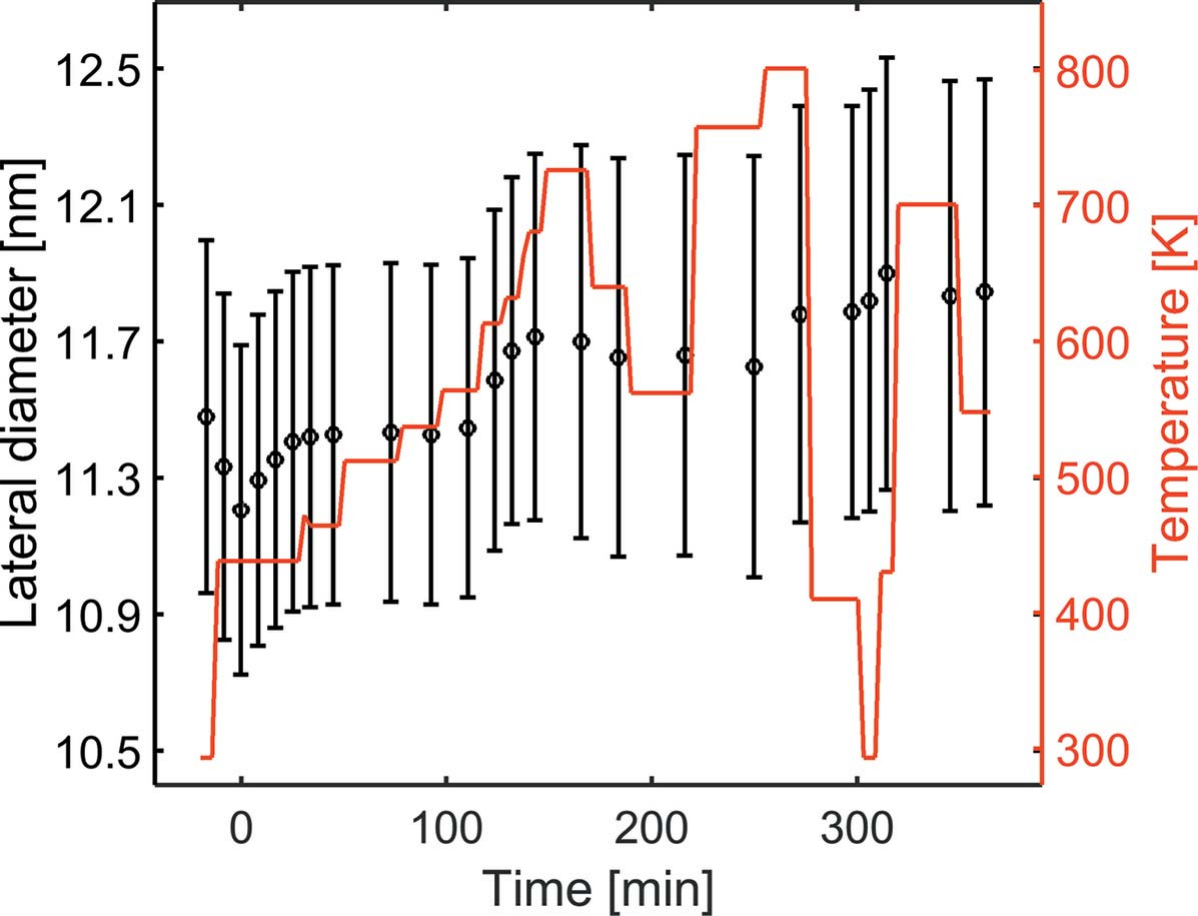
Lateral particle diameter (left axis) obtained from the fit during reduction in H_2_, first and second heating cycle in reactant gas of 1CO:2O_2_ plotted together with the temperature (right axis). The gas atmosphere in the reaction chamber was changed at *t* = 0 from H_2_ for reduction of the Ru particles to 1CO:2O_2_ for the CO oxidation.

The evolution of the lateral particle diameter during the reduction, the first and second heating cycle is shown in Fig. 10 ▸, the error bars showing the uncertainty of the fit. During the reduction in H_2_ at a temperature of about 433 K the lateral particle diameter decreased by about 0.3 nm. After changing the gas atmosphere to a mixture of 1CO:2O_2_ the particle diameter increased, in the first 25 min at similar temperature, by about 0.2 nm to 11.4 nm. At temperatures above 573 K a further increase of the lateral diameter to 11.8 nm was noticed. The standard deviation of the mean particle size remained constant at σ_D_/*D* = 0.16 during the experiment. The lateral particle diameter remained constant during the second heating cycle. The respective decreasing/increasing particle diameter in reductive and oxidizing atmosphere may be explained by reduction of the native RuO_2_ and re-oxidation of Ru. Alternatively, a difference in de-wetting/wetting of the particles due to higher surface energy of the Ru compared with RuO_2_ may be an explanation, or possibly a combination of both. To determine which, a combined analysis of the in-plane and out-of-plane GISAXS to resolve *H*/*R* of the catalyst particles is required, and will be possible in a modified version of the *in situ* micro-reactor flow cell with a sufficiently high exit angle in the future.

An increase of the scattered intensity at lowest *q* was observed at temperatures above 683 K, indicating the onset of particle sintering. At the same temperature we noticed the formation of RuO_2_ by the evolution of a broad RuO_2_(101) reflection in the GIWAXS pattern. At temperatures around 758 K the evolution of a sharp reflection at the peak position of RuO_2_ was observed, indicating the sintering of smaller RuO_2_ particles to large structures (see Fig. 8*b* ▸). The RuO_2_ crystallite size was determined to be *d* = 5 nm from the FWHM of the broad RuO_2_(101) reflection after the first heating cycle and correction for instrumental broadening. The sharp RuO_2_(101) reflection exhibits, however, a FWHM smaller than that estimated from the instrumental broadening which may be explained as follows. Though the mean coverage of the Ru deposition corresponds to 10% of a layer, the coverage was not homogeneous. The probability of particle sintering to form larger clusters depends on both particle mobility and on the surface coverage. In the case of an inhomogeneous spot of deposited Ru particles, the sintering may have occurred only locally in an area much smaller than the footprint of the beam on the sample, resulting in a sharper reflection than expected from the experimental geometry. The assumption that the investigated sample exhibited an inhomogeneous coverage and sintering occurred only in an area smaller than expected from the footprint of the incident beam is corroborated by the appearance of the sharp RuO_2_(101) and RuO_2_(211) reflections at a wavenumber *q* lower than expected from the lattice parameter [the RuO_2_(211) reflection is not shown].

The average crystallite size, neglecting the broadening calculated from the experimental geometry, was determined from the Scherrer equation, and assuming spherical crystal morphology, to be 37 nm from the sharp RuO_2_(101) reflection. This suggests that sintering of the catalyst nanoparticles includes the formation of RuO_2_, which has a higher mobility on the silicon oxide substrate due to its lower surface energy compared with Ru. At the same temperature an increase of the intensity in the horizontal line-cuts of the GISAXS was observed at the lowest *q* values (see Fig. 7*b* ▸), confirming the formation of large aggregates as observed from size-broadening analysis of the sharp RuO_2_(101) reflection in the GIWAXS. The model used for the analysis of the horizontal line-cuts of the GISAXS was therefore extended with a form factor for flat cylinders to account for island formation due to sintering. Due to the limited number of data points in the low-*q* region, which dominantly represents scattering of the larger islands, fits with the island diameter as a free parameter did not converge. Therefore the diameter of the islands was fixed to the crystallite size obtained from the width of the RuO_2_(101) reflection, and σ_D_/*D* for the islands was fixed to a similar value as determined for Ru clusters before sintering, and only the ratio of sintered to not sintered particles was added as an additional fitting parameter to the model. Analysis of the in-plane cuts of the GISAXS after the first heating cycle with this model indicates that the sample consists of approximately 0.5% (±0.4%) large islands with an approximated diameter of 37 nm. Sintering of Ru particles during oxidation reactions at similar temperatures and in excess oxygen, to form large islands, was previously observed by GISAXS (unpublished results achieved during beam time granted on proposal 20120493 at the cSAXS beamline at SLS) and was confirmed by high-resolution scanning electron microscopy.

**Figure 11:**
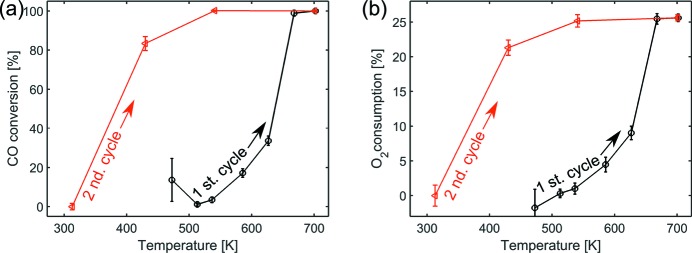
CO conversion level (*a*) and O_2_ consumption (*b*) during the first (black) and second (red) heating cycle.

The CO and O_2_ conversion levels, determined from the signal intensities of mass-to-charge ratios *m*/*z* = 28 and *m*/*z* = 32 during the CO oxidation are shown in Fig. 11 ▸. During the first heating cycle full conversion was obtained at temperatures between 633 K and 663 K. Full conversion of CO during the second heating cycle was already noticed at temperatures between 433 K and 533 K.

## 5. Conclusion and outlook   

We have demonstrated the feasibility of *in situ* experiments in a micro-reactor which combines the advantages of the high surface sensitivity of grazing-incidence scattering techniques, due to the low penetration depth of the incident beam in the substrate, with high sensitivity for the monitoring of chemical reactions due to small reactor volume. Combined with the mobile gas analysing system the micro-reactor flow cell permits the possibility of conducting grazing-incidence X-ray scattering experiments at any beamline with micro-focused beam capabilities.

As analysis of the out-of-plane GISAXS of the *in situ* micro-reactor in the current version is not feasible, we are working on an improved design with increased chamber height by selecting a SOI wafer with a larger device layer thickness and decreasing the width of the device.

For a better temperature control and more homogeneous sample heating, we will replace the hotplate by platinum strips directly evaporated on the back of the reactor. In combination with a four-pin connection the platinum strip can be utilized for both sample heating and temperature monitoring by resistivity measurements (Andersen *et al.*, 2012 ▸).
